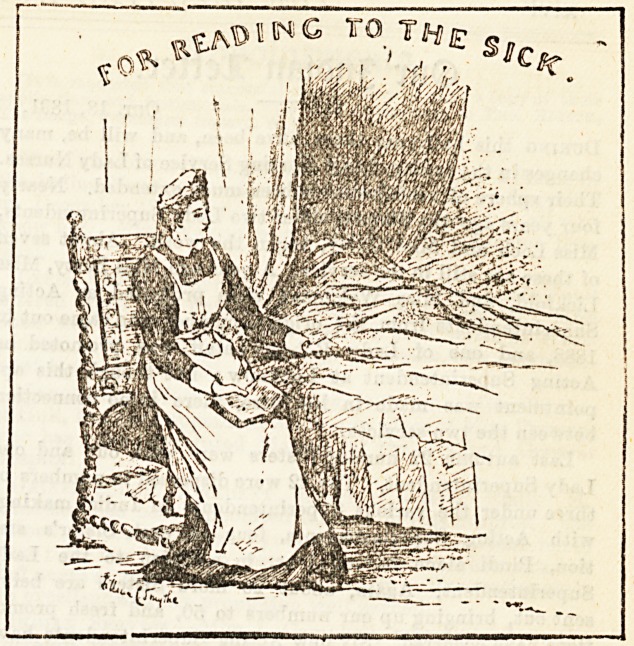# The Hospital Nursing Supplement

**Published:** 1891-11-21

**Authors:** 


### "The Hospital" Nursing Mirror.

Being the Extra Nursing Supplement of "The Hospital" Newspaper.

Contributions for this Supplement should be addressed to the Editor, The Hospital, 140, Strand, London, W.C., and should have the word "Nursing" plainly written in left hand top corner of the envelope.

### En Passant.

Northamptonshire Institution.—The fourteenth annual report of this Home is just to hand, and is cheerful reading, for it tells of the enlargement and alteration of the building satisfactorily concluded. The present staff numbers eighteen, and there are several probationers in training. Over 7,000 visits have been paid by the district nurses, and nurses have been sent to several villages where there have been outbreaks of fever. Altogether the Institution does excellent work, and is worthy of support.

A Nurses' Home for Aberdeen.—On November 6th a conference was held at Aberdeen to consider the necessity of starting an institute for private and district nurses: the Marquis of Huntly presided. The meeting agreed to the scheme laid before them, and a committee was appointed to carry it into effect. It is to be worked on the old lines: Probationers are to be bound for four years, and poorly trained and poorly paid; the public are to pay 25s. a week for a nurse's services, and the gains of the nurses are to be used to supply district nurses for the poor. We much prefer when these two lines of private and district nursing are kept separate, so that the district nursing is purely charity, which the public can be asked to support, and that the private nurses should be given a bigger share of their earnings. It is poor charity to take a nurse's earnings from her without her consent and give them to the poor. Miss Katherine Lumsden points out that already nurses for the rich are supplied by the hospitals of the town. In her opinion the crying want is cottage hospitals for the country districts.

Belfast News.—Two important meetings have taken place at Belfast lately, the chief being the quarterly Court of the Society for Providing Nurses for the Sick Poor. The report stated: "All summer the nurses have been kept so busy we have had considerable difficulty in arranging their work, so as to give them their much-needed holiday. They are this week attending 173 patients. In many instances several members of a family have been ill at the same time. The number of consumptive cases sent to us is very great. We can only take them in the last stage when they require actual nursing. I believe our nurses are often a greater blessing to the healthy members of the family than to the dying. The way in which phthisis is spread is deplorable, the very simplest sanitary precautions are ignored, the relatives appearing to think that the adoption of necessary care means want of affection or respect to the poor sufferer. The absence of fireplaces in so many of the rooms in which our patients live and sleep makes ventilation a matter of great difficulty in cold weather. We have twenty-five children on our books." The trustees of Dr. Johnston have given another £50 to the society. Then at the meeting of the Board of Guardians the question of the proposed increase of accommodation for the infirmary nurses came forward. There was a Mr. Morgan who got up and said they had done very well as they were for forty years, and he did not see why a nurses' home should be built; but his remarks were received with disfavour, and it was finally agreed that a new home is to be built, separate from the infirmary; so the nurses may look forward to shortly being made as comfortable as most of their sisters in English infirmaries.

Music as a Soporific.—A nurse suggests to us that when patients are first deprived of narcotics as they approach convalescence, and are irritable and sleepless, soft singing or gentle rubbing is advantageous, and induces sleep. We scarcely like to make the suggestion, for a nurse who sang out of tune, or with an untrained voice, would cause the patient simple agony; yet it is a fact that in this way only can the Guild of St. Cecilia do real good. Unfortunately, you cannot supply the guild to every sick-room by a tap which can be turned off or on at the nurse's discretion.

Thermometers.—In her last lecture of this session the Matron of the London Hospital announced that over 300 clinical thermometers had been broken in the wards during the year; in the previous year 581 had been broken; this shows why committees and matrons find it necessary to insist on care with regard to these fragile instruments. Private nurses are fond of indulging in expensive "half minute" thermometers which are exceptionally fragile, and almost bound to break in nursing a child's case. A sensible thermometer which rises in four minutes and is of stout make, is Bailey's 4s. 6d. thermometer.

Short Items.—Our readers should note the promotions in the Indian Nursing Service given in "Our Indian Letter"; they are of special interest in connection with the late awards of the Royal Red Cross.—Mrs. Grimwood has published a book giving her account of the Manipur incident.—Grahamstown Asylum (S. Africa) publishes a paper of its own; here is a hint for English attendants. We have a letter from Dr. Duncan Greenlees, which we will give next week.—The engagement is announced of Marguerite Norah, eldest child of the late Count Didier d'Ambléon, to Dr. Godfrey, of St. Thomas's. Miss Didier d'Ambléon trained at Birmingham, and has worked at Bath and Jersey.—The nuns of the Prague Hospital for Incurables were lately successful in extinguishing a fire which broke out in the building.—After twelve years of good work the Workhouse Infirmary Nursing Association is issuing a statement and seeking further support from the Guardians, whose infirmary system they have done so much to improve.—Miss Yeoman desires us to state that she has had general training at Bradford, as well as fever training.

Daily Nurses.—Any scheme for trying to supply trained nurses to the middle-classes is worthy of serious consideration. Miss Wood has just issued plans by which she proposes to send nurses for 10s. a-week to pay daily visits to patients, in the same way as the district nurses pay visits to poor patients. There are two points not fully explained in the papers before us; first, are the nurses fully trained who will be sent out; second, how can the nurse be the doctor's right hand if she only sees the patient for a few minutes' daily? Miss Wood writes: "I propose to create the daily nurse;" the phrase is amusing, especially when one remembers that a nurse by the day can be had from the Nurses' Co-operation, or to attend an operation, at exactly the same fee Miss Wood will charge, and that Miss Belcher and a few other nurses have for many years adopted this system of a daily visitation in slight cases. Still we are glad to see the scheme widening, for we know from experience what excellent work daily nurses can do, if they are thoroughly trained and specially adapted for the work.

### On Nurses and their Dress.

Ten years ago we seldom encountered a nurse whose outdoor garb distinguished her from other women. Deaconesses' Institutions and other religious Sisterhoods exhibited uniforms, but a nurse who wore any costume indicative of her calling, was, indeed, a rare sight.

To-day, things are different, and a morning's walk along Oxford Street or any other great thoroughfare, brings us face to face with "all sorts and conditions" of nurses. It is a pleasure to look at some of them, with their earnest, reliable faces, neat hair, and trim dresses clear of, not sweeping, the pavement; the graceful and serviceable cloak also of a reasonable length. The whole character of the nurse seems expressed as much by the perfect "fitness" of her costume as by the straightforward expression of the eyes which look so calmly ahead. This is the nurse of our dreams, the type we hope to secure when our turn comes for helplessness and suffering—though we trust that the hour of our need is far distant! But we shudder as we look at the next wearer of uniform who crosses our path, and note the loosely-tied bonnet perched on a towsled mass of hair (God help the patient of whose luxuriant tresses she has the charge!), the cloak fastened only at the neck, and blowing about like an ill-managed sail, revealing a broad expanse of apron, which if clean now, is in a position for entrapping a catholic assortment of unsanitary items for conveyance to the surgical patients, to whose bedside she is, perchance, about to return. The print dress, be it smart or shabby, is probably short in front, but long enough at sides and back to collect a neat border of road mud, or even worse, of the garbage of gutter or pavement.

Now, can these aprons and dresses be quite sanitary substances for re-introduction to the sick room? Even if the dress be of sensible shortness, the cloak is often just a little too long, and so reduces the skirt to its own bedraggled condition.

Sometimes it appears as if to wear a uniform reconciled a woman hitherto dainty and cleanly in her dress, to becoming a sloven. In home life the print dress is discarded if it becomes accidentally soiled, or even crumpled. In her nurse's life her daintiness should be accentuated, for her own sake, for her patients', and for her companions'.

Of the various untidy nurses who come before our eyes daily many have refined and sensitive faces, which make us think they must surely dress in darkened rooms, or they would never discredit their profession by appearing in such guise in the streets.

The frizzly curly hair which is so pretty on a young girl's head in a drawing-room looks unseemly when permitted to straggle over the rim of the nurse's bonnet. The heavy fringe of the Whitechapel girl is an offence to many besides their champion, Mr. Besant; but the nurse's rough head is a more serious matter. As a question even of ornament, a pretty face is but little improved, and certainly a plain one is made plainer by disorder. A neat head in a neat bonnet should be the rule, but as yet, alas! it is only the exception.

The cloak might fit well and entirely protect the dress and apron, and no garment should be long enough to make it's wearer the "street scavenger," to whom Ruskin likened the fashionable ladies, with their trailing skirts.

Of what avail are the surgeon's antiseptics when his helper the nurse is so inconsistent—she whose whole training is thus set at naught by her practice?

In major operations in hospital the Matron or the ward Sister makes sure that the nurse who is present is spotlessly arrayed, but the same care does not extend to those who are in daily attendance on all wounds. Surely lectures on antiseptics, the accurately measured disinfectants, the carefully kept dressings, and the jealously guarded sponges, are somewhat counteracted by the white apron and cuffs which have just made a journey in a closely packed omnibus.

True it is that "Evil is wrought by want of thought as well as want of heart," but there should exist no want of either in the woman who devotes her life to nursing.

She should be clean in her dress as she ought to be modest in words and ways. Serving in so noble a cause, fighting a constant battle against disease and death, she should scorn to give any occasion for light-speaking or ridicule.

To "see ourselves as others see us" would be a trying experience to most men and women, but risk this, dear nurses, and take heed to your looking-glasses; mark well what is reflected there, with somewhat of the cool criticism with which the general public regards you; and then it is probable that the draggle-tailed nurse with the wild looking head will soon cease to exist.

In her stead the delightful typical woman will reign, and the sight of the figure in nurse's uniform will be no longer what it is now.

Is it indifference or vanity which has developed the present style? There must be a reason for a state of things so opposed to all cleanliness and common sense. Whom does it please? Not the doctors, nor the Matrons, nor the public. Of this we make emphatic assertion. Then is it to please themselves that our nurses have fallen into these ways, thoughtlessly risking for themselves the many dangers which follow on damp skirts and stockings, as well as the many risks which they thus secure to their patients?

The Nightingale probationers are forbidden to wear their ward costumes outside the hospital, which is a simple way of avoiding a difficulty, but we incline to think that ordinary prudence on the part of nurses would obviate the necessity for such a rule becoming universal, and we should be sorry indeed to see the pretty uniforms banished from the streets because the mode of wearing them is not always a wise one.

### A Nurse in Natal.

It is thoroughly understood in England that no service rendered to the sick is considered menial or unbecoming, irrespective of the sex of the patient. The nurse is trained to consider that the loftiness of her calling sanctifies every action for the benefit or ease of her patient, and that decency is studied, not by the avoidance of certain duties, but by the nice and considerate discharge of them. Miss Goodricke had, however, to learn that different ideas and manners obtain in different lands, and she was surprised to find that (except the sponging of fever cases) no nurse was allowed to wash a male patient, however helpless, or to dress his bed sores, however severe. She was told it was "not decent." These services, requiring, as they do, such delicate care, and tender, womanly skill, were always left to the coolie attendant, and neither Matron nor nurse must ever be present at their performance.

It may be doubted, I think, if the ministrations of these men, who, though naturally quick, were totally untrained, could take the place of a woman's gentle attendance without some detriment to the poor invalid's ease and comfort. It must, therefore, be supposed that the comfort of the patient was made subservient to a colonial sense of "decency." During Miss Goodricke's engagement at White's Hospital, there was one case which appealed especially to her interest and sympathy. It was that of an elderly man, suffering from a fatal injury to the spine, which rendered him completely helpless. This poor man, during the latter months of his life, suffered terribly from bed sores, and it grieved his nurse to hear him complain, as he sometimes did, of the rough, untender care of his coolie attendant. She soon begged that she might be allowed to dress his back herself, but, notwithstanding it was well known that this poor invalid's days were numbered, her request was denied on the usual ground of decency. "Might she not then stand behind the screen during the coolie's performance of his work, so that she might at least direct him by word?"

This also was refused; "it was against the custom of the institution."

It must not be supposed that there was any want of ordinary kindness shown in this hospital. I have gathered no such impression, either from my friend's letters, or from my subsequent inquiries when visiting the colony. A doctor is, of course, satisfied if he sees his actual orders carried out; it is for the nurse personally to understand how much a patient's comfort may be increased, and his sufferings relieved by the individual attention, at once skilful and tender, of his attendant. While upon the subject of decency, I may mention that only in cases of admittedly serious illness, such as fever or dysentry, were the nurses allowed to attend to the private wants of their patients, all utensils used in the wards being removed and cleansed by the coolie servants. This work is always considered an important part of the nurse's duty in England, and justly so, for how otherwise is the doctor—through the nurse—to be made acquainted with the earliest symptom of some fresh complication in a complaint not perhaps at first considered serious? At any rate, I have often heard it remarked, by persons who have visited the Myburg Hospital, that they could wish greater attention bestowed on this detail, if not for the patients' good, at least for the comfort of visitors.

I mentioned some time ago that Miss Goodricke found the management at White's Hospital very lax as compared with that of establishments in this country. As an instance of this I may quote the case of a man brought into the hospital one afternoon about four o'clock.

He was a transport rider, and had recently been exposed to the heavy summer rains, which had caused his present illness. At first it was not thoroughly understood from what special complaint or form of cold he was suffering, but he was put to bed, and made as comfortable as possible pending the doctor's visit next morning. About seven o'clock, feeling sure from the patient's heavy breathing that this was a case of pneumonia, Miss Goodricke repaired to the Dispenser to request that he would visit the ward, as she could not but think that speedy remedies were necessary.

This gentleman, however, was just going to his dinner, and thought the patient could wait better than himself.

An hour and a-half passed before he appeared at the bedside, and then he ordered no more stringent measures than the application of linseed poultices. This was, of course, done; but at eleven the same night the patient died. The details of this case were probably never known to the visiting doctor, who was a clever man, of high reputation in the colony, overweighted with work; and as the responsible actor in them is now removed to another sphere, I mention them to show how conscientious each member of a hospital staff should be in the performance of their duty.

(*To be continued.*)

### A Bed for a Sick Nurse.

We have to acknowledge the following further subscriptions towards endowing a bed for the use of sick nurses at the Brassey Home, St. Leonards-on-Sea. Mrs. Carr-Gomm, one guinea; Nurse Groom, 5s.; Miss Rosling, 5s.; One of the Second Thousand, 6d.; Nurse Scott, 2s.; Nurse Danter, 10s. 6d.; A Manchester Nurse, 2s.; collected by Nurse Franklin from her fellow nurses, 9s. 6d.; making a total of £30 18s. 6d., and leaving 12s. still needed, or better still, one more guinea subscription. Please do not be afraid to send, for any odd sum over can be used to help furnish the room.

### THE RAINBOW.

There is nothing which helps us on better in life than a hopeful disposition, and we can foster and encourage this until it becomes natural to us. All tasks are easy if we feel they will be crowned with success, and trials and sufferings light, with hope to cheer us on our way. It is the same all through life; "hope on, hope ever," should be our motto. But more especially so is it in sickness. Our tears have fallen on our path, for we are grievously tormented by pain, and are lying sick, nigh unto death. Are we forsaken of God and man think you; is there none to rescue us from our hapless plight? Look up into the heaven and see the rainbow in the cloud! The very waters which seemed as if they would destroy us have become the sign of our safety. God has promised that they who flee to Him for help shall be saved. There are many kinds of Hope. For instance, the "forlorn hope," which prompts the soldier to desperate deeds when he has arrived at his last resource. There is the hope which so cheers one suffering soul that notwithstanding throbbing brow and aching limbs he pulls through successfully to health and strength; while another, lacking it, sinks slowly out of life.

There is an old saying which nearly always proves a true one, that a rainbow in the morning is the shepherd's forewarning; but a rainbow at night is the shepherd's delight. As the shepherd is prepared by the early bow for storms further on in the day, so we may learn that a prosperous happy youth is frequently followed by crosses and trials which are sent to remind us this world is not our abiding city. And though our day of life may have been dark and dreary, yet when our course is nearly run, there shall be light to cheer our parting and declining steps.

The world once nearly perished by a flood of waters, but from the same drops which came to destroy, God formed the lovely bow and placed it in the Heavens as a token of His pardon and mercy. Ever since the rainbow has been the symbol of hope, and we may take heart and comfort ourselves with the certainty that the world, your world, my world, everybody's own little surroundings are safe when under the shadow of God's arch. We will lay hold of this hope for it maketh not ashamed. With Christ's blessing shining on us, our tear-drops and our afflictions will sparkle like jewels in the beams of the sun of righteousness. The glorious rainbow

"Flingeth soft radiance far and wide,
Over the dusky heaven and bleak hill side,"

satisfying the heart and eye with beauty, while brighter even than the rainbow is the cheerful heart which throws the soft gleam of Christian love and hope on all around.

### Our Indian Letter.

Oct. 18, 1891.

During this half-year there have been, and will be, many changes in the Indian Army Nursing Service of Lady Nurses. Their sphere of usefulness has been much extended. Nearly four years ago ten Sisters, under two Lady Superintendents, Miss Loch and Miss Oxley, began the work. About seven of these are still in the service. A year ago Miss Betty, Miss Lickfold, and Miss Welchman were promoted as Acting Superintendents from the original number who came out in 1888, and one of Lady Roberts' staff was promoted as Acting Superintendent at Lucknow; but though this appointment was made in her case, there is no connection between the two services.

Last autumn 22 nursing Sisters were sent out and one Lady Superintendent. The 22 were dispersed in numbers of three under the various Superintendents in India, making, with Acting Superintendents, four at each Sister's station, Pindi alone having four, in addition to the Lady Superintendent. Again, about 20 more Sisters are being sent out, bringing up our numbers to 50, and fresh promotions have occurred. Six new Acting Superintendents have been made, and five new stations created. This is an extract from the *Gazette of India*: "General Orders by His Excellency the Commander-in-Chief. Head-quarters, Simla, 12th October, 1891.—The Commander-in-Chief has been pleased to make the following promotion in the Indian Nursing Service, with effect, from 1st October, 1891: As Lady Superintendent—Miss A. R. Betty; as Acting Superintendents—Miss E. D. Harris, Miss M. E. M. Latch, Miss M. S. M. Beresford, Miss D. M. Moore, Miss M. J. Hislop (vice Miss E. Welchman, resigned Acting Superintendent), Miss M. A. Wildman." Miss Harris, Miss Latch, and Miss Beresford came out in 1888, Miss Moore and Miss Hislop, October, 1890, and Miss Wildman last Christmas. Miss Moore and Miss Wildman are members of the National Pension Fund for Nurses, Miss Wildman being one of the first thousand who was present at Marlborough House July, 1890.

As so many fresh recruits are coming out to us this trooping season, I venture on a few hints that may be useful to them, from one who is already out here. For convenience sake I will address them in the second person: Dear sisters, Government will offer to advance £20 out of your future pay; if you can manage without the advance, do, for they also allow £15 for outfit-money. You are not obliged to spend all that at Harvey Nicholls; any bank will cash the Government draft. You will need 18 aprons, white linen or twilled cotton, calico or nainsook, will do; they are made plain, with two pockets and square bibs, bands buttoned or invisible strings. H. N. last year put sashes or broad strings on the aprons, which were not uniform. Harvey Nicholls have all the patterns, and they can be seen there; the bonnets you must get there, the coats it would be advisable also. Get six pairs of regulation bonnet strings, and bring out as much grey ribbon as will re-trim your bonnets when necessary, and frilling for the bonnet-caps. If you buy one grey dress and have it made, it might do, and get material at H. N.'s, and have the other dress made up at home. I brought out, in addition to two grey dresses, material for a third, and two dozen regulation buttons. I found I had saved by doing it; you can't get the serge here. I brought out six white dresses, and got six more here; they are cheaper here. £5 will be enough ready money to bring out with, you. Your mess bill on board ship should not exceed £3, at 2s. per diem, and you may wish to spend a little money at Malta and Port Said. When you reach Bombay, go to the District Staff Officer and draw your first month's pay, it will give you money to go on with, and will not be counted in the money that may have been advanced to you in England, that will be subsequently deducted from the next following five months pay, and, of course, you will lose on the exchange.

In buying boxes, have tin trunks principally; have one large wooden one, tin lined, for the hold; medium sized tin boxes are more useful than large ones, flat wide ones best, two moderate sized tin-trunks, one for "present use" baggage room, and one for "change of clothing" at Suez, one good sized tin cabin trunk, one large Gladstone bag for cabin, and one hold-all; these I bought very reasonably at Baker and Sons, High Street, Holborn; leather boxes are more expensive, and get destroyed out here. Have your name painted on each box in front, not too large, "Miss E. Browne, I. N. Staff," or "Indian Nursing Staff," or "I. N. S.," just as you like, any of the three titles will do. No padlocks allowed. For your tin trunks buy leather straps, cords are not allowed, and if there are no straps the locks burst. The wooden box "heavy baggage," for hold, have screwed down, four screws will do; for "light baggage" concave-topt trunks are allowed; you will get all information gratis, about regulation sizes, &c., from Curtiss and Sons, Portsmouth, whom I advise you to employ as agents; you can store your boxes there, they will meet you at Portsmouth, put everything for you on board in its proper place, and all for a few shillings. I employed them and their corresponding agents in Bombay, King, King, and Co. Some of my luggage I never saw for a fortnight before I left England till I arrived at my destination in India, and nothing lost.

Don't buy a travelling or other kind of bath, Government supplies each Sister with a regulation tub; if you have one you can bring it, if not, buy an extra trunk by preference. At Portsmouth buy a soiled linen bag with lock, about 4s. 6d. or 6s. at Curtiss and Sons, also a deck-chair, which latter, have your name painted on or you will have it stolen.

All nursing Sisters are immediately under an acting Superintendent. She provides the table-linen, knives, forks, spoons, &c. Cooking utensils and crockery, for the use of which the nursing Sisters on arrival at a station pay their share of the expenditure, or a sum is charged them with the housekeeping account, monthly. The Acting Superintendent, whose authority over the nursing sisters is supreme, does the housekeeping, selects and engages the servants. Monthly expenses, according to the station you are in, vary from 35 rupees to 90 rupees per month. Each Sister has to provide her own bedding and bed linen. Government supplies spring beds. My bedding consists of no mattress, a resai, which you can buy at Bombay for 2s. 8d., or 3 rupees, it is like an eider-down, only more substantial, 7 ft. by 4 ft.; two wool pillows—feather pillows are most expensive out here; six pillow-cases, six thin cotton sheets, two blankets, *i.e.*, a double one cut in two; and two counterpanes, these would be cheaper in England; also bring out bath and huckaback towels.

About 50 yards of art muslin, at about 2d. per yard, would be a most useful thing to bring out; it would save you buying muslin curtains here at ten times the expense. I required for one room I had six pairs of curtains. Bring plenty of gloves in a glass bottle, white and grey being the most useful colours for you. Suede and silk are very nice, the latter are very dear here, silk gloves being 2 rupees 8 annas a pair; that would be almost 5s. at home. White kid gloves, 4 rupees 8 annas a pair—that is about 9s. at home. Tan gloves don't look bad with uniform. A warm wrap or cloak is very necessary—furs get mouldy out here—an imitation astrachan cape and muff would be useful, grey would match uniform. One evening dress is quite enough, as you can only wear it when on leave. The Sisters when dining out usually wear white silk uniform dresses with red velvet belts and facings; at one station they wear cashmere. You will need a sun hat; that would be got cheapest here, and the Sisters at some stations wear, with uniform, white sailor's hats with white ribbons. These have been sanctioned for nearly the whole service for tennis, and when the bonnet is not suitable. Brown leather shoes and slippers look best with summer uniform. You will need more slippers than shoes, for you will find shoes or boots too hot in summer. Bring out pyretic saline, good for the sea voyage, and as a preventitive of boils in the hot season. If you wish to go out of uniform when on leave, a good dress to have is blue serge and jacket to match, with several shirts to vary your costume, a felt hat, and a sailor's or a light straw hat. A smart thin dress for garden parties and gymkanas, and an evening dress that can be high or low, ball or dinner gown. Don't bring out too heavy a riding-habit. It is well, if you ride, to bring out your saddle and bridle, &c.; they are dear out here.

On the troopship be agreeable to all, but don't be frivolous or fast; it gives the service a bad name, and your character will be well discussed on board ship, and whatever reputation you make for yourself there will stick to you; the officers and their wives who have been with you there will be dispersed over India, and you will meet them again in all parts. If you have met their approval, you will be made much of; but if you are "Miss Blank, who made a dead set at young Bow-wow, of the 150th," or "Miss Stiff-neck, who made herself so disagreeable in the ladies' cabin," you will be nowhere—you will be ignored. Keep out of quarrels, and don't take sides; don't flirt; act up to your profession. When I arrived in Bombay I heard all the gossip regarding the Sisters who had arrived before me. One officer, high in rank, after visiting the Staff Office, came back and told me some of the *amusing* little anecdotes he had picked up there. Hoping my few hints may be of use to you, and that I shall have the pleasure of welcoming a few of you soon, I remain, yours truly,

An Old Fogey.

### The Nurses' Bookshelf.

### A MANUAL FOR NURSES.*

This is a small book of prayers for nurses, rather fuller than the volume issued by the same publishers some years ago. It is eminently high church, with forms for confession, &c., and we should not recommend it to the members of the St. Barnabas Guild even had they not a manual of their own. We are rather doubtful whether set prayers before an operation, after laying-out the dead, &c., are desirable, but possibly some may find them helpful. A nurse's whole life ought to be one long prayer "expressed in deed," the necessity for perpetually seeking expression in phrases, is, we believe, only felt by a few.

### TWO OLD FRIENDS.†

We have some diffidence in praising the next two volumes sent for "The Nurses' Bookshelf," because they are issued from our own office; but luckily the fact that both have been so far appreciated as to have reached a second edition makes praise superfluous. Dr. Lewis's book is considerably enlarged and improved; it has numerous illustrations, including representations of all the chief instruments a nurse meets with in a surgical ward. The "Case-book" is now arranged so that each page holds a week's record instead of a fortnight's; this leaves more room for the figures. It is also in a cheaper edition now, and can be had post free for 4s. 6d. per dozen.

* "The Servants of the Sick." Masters and Co. Price 1s. 3d.

† "The Theory and Practice of Nursing," by Dr. Percy Lewis. Second Edition. Price 3s. 6d. "The Nurses' Case Book." Price 6d. The Hospital, Limited.

### Appointments.

[It is requested that successful candidates will send a copy of their applications and testimonials, with date of election, to The Editor, The Lodge, Porchester Square, W.]

Miss Florence Walton has been appointed a Sister in the Indian Nursing Service, and sails for India by troopship on December 12th, followed by the good wishes of all who know her. Miss Walton was trained at Westminster Hospital.

Leeds Fever Hospital.—In September Miss Annie Bond entered upon the duties of Sister at this Hospital. Miss Bond was for two and a-half years a staff nurse at Westminster. We regret that, through a mistake, this announcement has been delayed.

Manchester Hospital for Consumption.—Miss Bower, of Hull, has been appointed Matron in place of Miss Farmer, who has been appointed Matron of the Stafford General Hospital.

Gainsborough Accident Hospital.—Miss Emily H. Windeler has been appointed Matron of this hospital; she trained at Newcastle-on-Tyne, and become assistant nurse there; for the last two years Miss Windeler has acted as staff nurse at the North Riding Infirmary. Her testimonials are excellent and her promotion well earned.

Indian Service.—Miss I. Campbell, who trained at the Northern Hospital, Liverpool, and is still on that nursing staff, has been appointed a Sister in the Indian nursing service.

Zenana Medical College, London.—Miss I. Prosser, who trained at the Northern Hospital, Liverpool, and is now a Sister at Marylebone Infirmary, Notting Hill, has been appointed Matron of this Medical College.

Royal Hospital for Diseases of the Chest, City Road.—Dr. S. Herbert Habershon, who has been for the last five or six years one of the Assistant Physicians at this hospital, has been recently unanimously elected one of its Physicians.

### Presentation.

When Miss A. Browne resigned the Matronship of Manchester Royal Infirmary, she was presented by the St. Barnabas Guild, of which she was local Superior, with a handsome crucifix. At the foot was a silver plate with an inscription stating the occasion of the gift.

### Notes and Queries.

To Correspondents.—1. Questions or answers may be written on post-cards. 2. Advertisements in disguise are inadmissible. 3. In answering a query please quote the number. 4. A private answer can only be sent in urgent cases, and then a stamped addressed envelope must be enclosed. 5. Every communication must be accompanied by the writer's full name and address, not necessarily for publication. 6. Correspondents are requested to help their fellow nurses by answering such queries as they can.

### Queries.

(13) What is consumption of the bowels? What causes it? What, are the chief nursing points?

(14) What is Friar's Balsam?—*C. E. J.*

### Answers.

(12) We know of no home which will take the case you mention, though you might try for admission at the Hospital of St. Elizabeth and St. John, Great Ormond Street, W.C. Also some of the cottage hospitals will occasionally take an incurable case, the St. Peter's Cray Cottage Hospital for instance.

*Darum.*—Please send name and address, or we cannot use your story.

*A Sympathiser.*—Your egoistical and personal letter can be of no interest to our readers, so we suppress it.

*B. C. M.*—Apply to the Director-General, Army Medical Department, Whitehall, S.W. You must have had three years' training, or it is useless to apply.

*Emmeline.*—You must be quite strong before you take up nursing even in a children's hospital. A tired, weak nurse could not possibly help to "cure" children.

*"A Pro."*—Quain's "Anatomy" and Huxley's "Physiology" are two good works to study. No, you are not too young for a children's hospital, *if* you are sure you are quite strong.

*E. P.*—We wish you would read Andrew Lang's lecture on "How to Fail in Literature," and mark his sarcasms on poems beginning "Only." You must practice a great deal before you can write verse worth printing. Both rhymes and metre are faulty.

*Christmas Competitions.*—Parcels received from Nurse S. E. Parker and Elizabeth Bishop.

*Birmingham City Asylum.*—Your news is held over so that an illustration of the medal may appear.

### "Dr. Sutters."

When stray rumours of the probable retirement of Dr. Kingdon from his practice in Hook-in-the-Vale were confirmed by that excellent quidnunc Miss Gravestock, the post-mistress, folk began to wonder who would fill his place. Few changes diversify the set monotony of Hook. Now and then a child is born, or a labourer meets with some casualty, or one of the old people at the almshouses "goes off with rheumatics," all of which events are turned into pleas for wasting time in gossip at the general shop and the Farriers' Arms; but, except in birth or death, no one comes and goes at Hook. It was, therefore, a time of excitement when later reports from the Post Office announced that Dr. Kingdon had found a successor in a gentleman of the name of Sutherst.

"Med he be older or younger nor Dr. Kingdon, ma'am?" "D'ye know if he's a married man?" such were the queries that daily assailed Miss Gravestock when the wives and daughters of the village went to the post with the perennial order for "a penny stamp and a ha'porth of writing-paper."

"I should be saying more than I know if I told you any more than that the new doctor's name is Sutherst," was Miss Gravestock's answer. "That I do know, for there's a letter waiting here for him."

A few days after, a middle-aged gentleman, with a stoop and gold-rimmed spectacles, was seen walking up the street with Dr. Kingdon, and Hook was apprised that "Dr. Sutters" had come. He was not much of a man to look at after all. He was pale and weakish, and his clothes were not spruce enough to command reverence. The combined discernment of the parish was not able to "make much out of him," and, to tell the truth, the women folk were distrustful because of his physical conformation. It was certainly a trifle hard upon the little man that he should have to run the gauntlet of such bucolic criticism before he had attended a single case, or even spoken to anyone save his predecessor. Neither was it fair of Hook to impeach his acumen because his height and girth were below the burly Dr. Kingdon's. But of such is the crude judgment of the rustic mind. And then the hat—that badge of gentility in the country—was not of the chimney-pot shape, but of unfashionable and well-worn flexible felt. So, in the same way that Hook distorted the new doctor's name, it hastily pronounced an adverse opinion upon his attainments; which perversions are quite normal to the village, and understood of those who know the intellectual temper of its inhabitants.

"You will call on the people," said Dr. Kingdon to his successor.

"Of course, if I'm sent for," he returned.

"I see you don't quite understand," explained Dr. Kingdon. "It is customary in the country to look in now and then at the bigger houses in a friendly sort of way."

Dr. Sutters looked somewhat troubled. "Yes, I see," he remarked. "I daresay I shall do as you say; but I'm not sociable, I'm afraid. I could never make an income worth mentioning as a medical man. I can't talk to women."

"Ah, that's unfortunate," murmured Dr. Kingdon, looking with raised eyebrows at the odd figure by his side. "But you are joking, sir, or else it's modesty."

"No, indeed," stuttered Dr. Sutters, "it's plain truth. I am not a misogynist—on the contrary, I admire the sex—but I repel them. Physically, I think I know a little about women's nature—you have seen my articles in the medical journals?—but, mentally, I confess they are riddles to me."

"Oh! well, of course, we all admit that," laughed Dr. Kingdon.

"It is more to my inclination to write than to practice," continued Dr. Sutters. "Here I shall enjoy that quiet which is so essential to good literary work. I have heard, too, that this neighbourhood abounds with beetles."

"With beetles?"

"Yes; I am writing a work on the Coleoptera. It will be in two quarto volumes, with coloured plates. It is really on account of the beetles that I have decided to settle here. I have private means, as I told you in my second letter. It's very fortunate, for I have a poor bedside manner."

In a short time the Hookites found out that the way to win Dr. Sutters's good favour was to take him beetles. It was a source of wonder that a crawling thing should cause the taciturn doctor to manifest such joy. Tom Manning, the blacksmith, was never weary of rehearsing the extraordinary rapture that "a nasty greeny animal" produced upon Dr. Sutters. "He guv me a shillun for it!" he would declare.

"He's a wunnerful deep scholard in the ways o' them critters," said Barnabas Endacott, the fogger. "He told my gaffer all about them Grub-unders as does such mischief to the turnups. Howsomever I'd sooner see him take a gun and go arter the pheasants, like Dr. Kingdon. 'Tis a niggly sort o' way o' spendin' time to go poking round trees and dabbing in ponds for these beedles. What is a beedle when you've cotched it? That's the pint I should like 'e to settle, Tom Manning."

The higher strata of Hook society also regarded Dr. Sutters as very eccentric. The Vicar met him in the road one day, and after introducing himself, invited him to dine at the Vicarage.

"Thank you, I'm extremely obliged," said the doctor. But he was glad that no day was fixed, for the Vicar had three unmarried daughters. The eldest, a dark girl of about five-and-twenty, always passed him with premonitory symptoms of a disdainful smile upon her face. How well he knew that expression! It spoke of suppressed giggles. Young women always looked at him in that way.

"I suppose I must be a comical looking fellow," he mused. "My nose is not normal, I know. Perhaps it's that which affords them inward mirth. But there is something more than a sense of the ridiculous in that girl's face; there is positive aversion."

He asked his housekeeper what they called the eldest Miss Norton.

"Mabel, sir. A fine young lady, don't you think? She's had more than one offer, I've been told, but she's one of the independent sort. Good family connections, you see, sir."

"Ah!" he said, as he spread out a beetle's legs on cork and fixed them into position with pins.

(*To be continued.*)

### Amusements and Relaxation.

**SPECIAL NOTICE TO CORRESPONDENTS.**

**Fourth Quarterly Word Competition commenced October 3rd, ends December 26th, 1891.**

The word for dissection for this, the EIGHTH week of the quarter, being

"LIFEBOAT."

| Names. | Nov. 12th. | Totals. | Names. | Nov. 12th. | Totals. |
|---|---|---|---|---|---|
| Lightowlers | 44 | 278 | Jenny Wren | 39 | 198 |
| Bonne | 42 | 298 | Darlington | 39 | 277 |
| Morico | 48 | 327 | Nurse G. P. | — | 99 |
| Robes | — | 143 | Hetty | 35 | 192 |
| Dulcamara | 45 | 290 | Janet | 35 | 233 |
| Psyche | — | 7 | Jackanapes | 43 | 220 |
| Agamemnon | 46 | 310 | Ex Nurse | — | 99 |
| Nurse J. S. | 36 | 275 | | | |

**Notice to Correspondents.**

All letters referring to this page which do not arrive at 140, Strand, London, W.C., by the *first post on Thursdays*, and are not addressed PRIZE EDITOR, will in future be disqualified and disregarded.

## Figures and Tables

**Figure f1:**